# Virtual Trauma Meeting as a Component of Undergraduate Orthopaedic Education

**DOI:** 10.7759/cureus.26810

**Published:** 2022-07-13

**Authors:** Rosalind Di Traglia, Sally Rankin, Jonathan M French, Kathryn Ecott, Henry Burnand, Fergal Monsell

**Affiliations:** 1 Trauma and Orthopaedics, Bristol Royal Hospital for Children, Bristol, GBR; 2 Trauma and Orthopaedics, Bristol Royal Infirmary, Bristol, GBR

**Keywords:** covid-19, medical student education, virtual education, trauma meeting, trauma and orthopaedics

## Abstract

Introduction: Virtual teaching has proven effective for medical students during the COVID-19 pandemic. This study is the first to describe an undergraduate orthopaedic teaching strategy in the format of virtual trauma meetings (VTM).

Methods: Clinical medical students from the Universities of Bristol and Cardiff were invited to attend five VTM between October and November 2020. These were delivered by consultants and speciality doctors via Zoom software. An 11-item feedback form was distributed after each session to assess the relevance of teaching material, student confidence in asking and answering questions, and if students would benefit from further sessions. Several open-ended questions were designed to evaluate aspects of the session that were most useful, which orthopaedic topics were of high priority and if they had any suggestions for improvement. Our initial aim was to assess student acceptance of the virtual format. Several months later pre-recorded material was uploaded onto YouTube and post hoc questionnaires were analysed.

Results: A total of 50 students attended, with a median of 11±6 attending per session, producing a total of 26 feedback responses. Among the responders, there were 10 males and nine females and 63% of the students were in their third year. 100% of students felt comfortable asking questions and 96% felt comfortable answering questions. X-ray interpretation and management of fractures were the highest priority subjects. The majority of students considered the interaction between senior and junior doctors most valuable, and the most common improvement suggested was the inclusion of polls or OSCE-styled questions.

Conclusions: VTM could be a useful resource to enhance undergraduate trauma and orthopaedic (T&O) education by providing student-focused material in an open learning environment.

## Introduction

The COVID-19 pandemic marked a paradigm shift in undergraduate medical education worldwide, towards blended teaching, a modality that adopts virtual and face-to-face formats. Increased interactivity, self-directed learning, and flexibility are among the many advantages of this approach. Virtual software with chat functions can engage diverse learner types, sometimes anonymously and as effectively as in-person [1,2]. Pre-recorded videos can consolidate live material and increase knowledge retention relative to traditional lectures [3]. Several studies have determined that e-learning is cost-effective and acceptable to orthopaedic trainees but have not evaluated undergraduate virtual trauma meetings (VTM) to the same effect [4-6].

Trauma meetings are daily team briefs dedicated to patient handover, organising theatre lists, and educating trainees [7]. VTM facilitate synchronous case-based discussions with junior and senior doctors in a student-focused manner and whilst they were feasible during the pandemic, their educational potential was not explored [7].

Our aim for the virtual meetings is to substitute teaching for students who missed orthopaedics education secondary to the pandemic, enhance core teaching and act as a revision aid for final year medical students. Our objective is to evaluate the initial student acceptance of VTM in the context of COVID-19. We anticipate this strategy will enhance undergraduate trauma and orthopaedic (T&O) education in an open and adaptable setting.

## Materials and methods

The sessions were advertised to Bristol and Cardiff medical students in years 3-5 by the Bristol orthopaedic society Facebook group and medical school newsletter. Five meetings were delivered bi-weekly between October and November 2020. Sessions took place on Zoom® from 20:00-21:00 to accommodate daytime teaching and maximise attendance. The meetings featured panel discussions between consultants and trainees using radiographs and clinical images of regional fractures. Anonymised patient cases were selected by orthopaedic consultants and ratified by our orthopaedic society representative in accordance with the undergraduate curriculum. The live meetings were recorded and edited into 10 15-minute cases using iMovie®. Following peer-review by university faculty, these videos were uploaded onto YouTube and Bristol’s undergraduate blackboard website, to increase accessibility (see Table 2 in appendix).

Certificates of attendance incentivised student responses and post hoc questionnaires designed in Google forms were used for retrospective assessment of student satisfaction. This required identifiable information, which we emphasised would only be used for distributing certificates and anonymised data analysis. The survey evaluated student satisfaction, the programme’s relevance, limitations, and most useful aspects. Three questions required binary yes/no responses, but the majority were open questions designed to gauge the student perspective. Responses were analysed several months later after pre-recorded materials were uploaded onto university pages. Post hoc analyses included descriptive statistics and thematic analysis for binary and free-text answers, respectively.

## Results

A total of 10 trauma cases were delivered in five VTM to a total of 50 year-3-5 medical students from Bristol and Cardiff University. Four of the five meetings obtained feedback responses as the first was a proof of principle pilot. There was a median of 11±6 participants per session, with a survey response rate of 50% (±62), producing 26 feedback responses overall. Three of the survey respondents were in years 4/5, there was one gateway student and twelve were in their third year (Table 1). There were two repeat attendees, both in their third year of medical school.

**Table 1:** Student feedback questionnaire

Questions	Student responses, n (%)
University email address	
Year Group	
Year 1	0
Year 2	0
Year 3	12
Year 4	3
Year 5	3
Gateway	1
A. Was teaching targeted at your level? Y/N	19 (100)
B. Did you feel able to ask questions? Y/N	19 (100)
C. Did you feel comfortable answering questions? Y/N	18 (96)
D. What aspects of the session were particularly helpful?	
E. What suggestions do you have for improvements?	
F. What topics would you be keen to cover linked to the undergraduate curriculum?	
H. Did you find the learning points from the starter questions useful for enhancing your curriculum learning?	
Will you attend again? Y/N	19(100)
Would you recommend this to a friend? Y/N	18(96)

**Figure 1:**
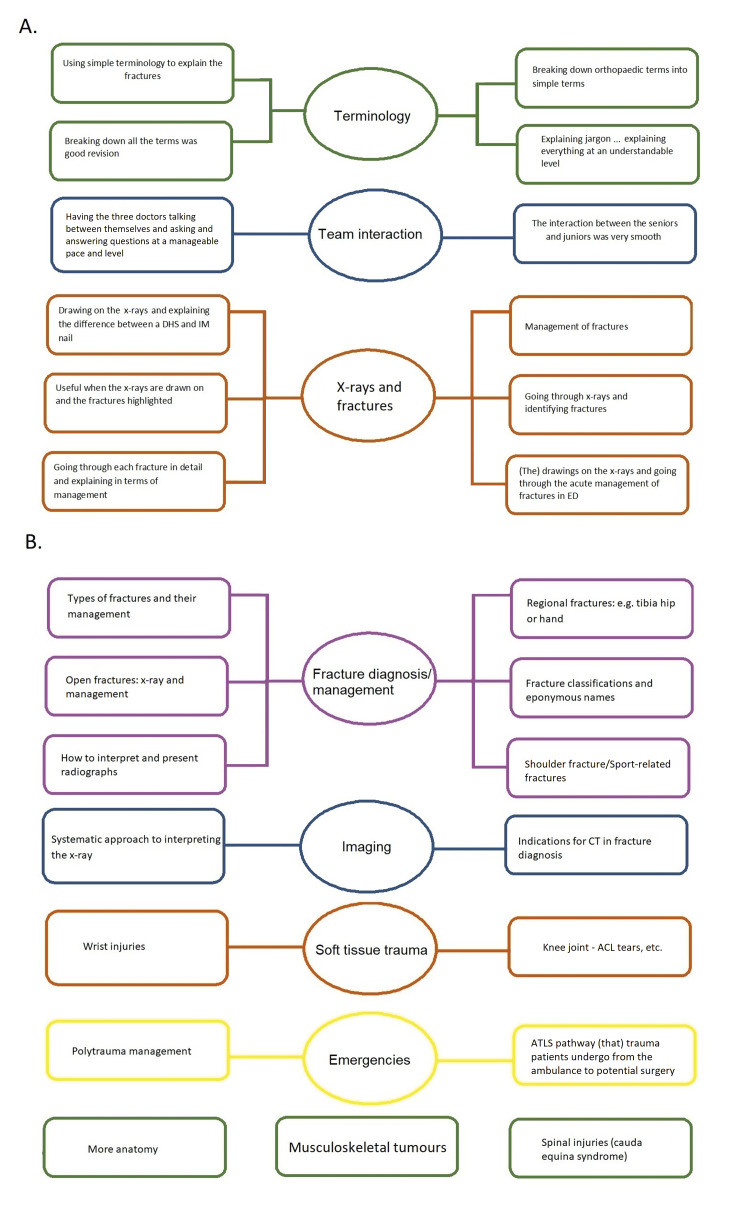
Thematic tree produced from thematic analysis of qualitative data. Direct quotes of students made over the four sessions are detailed in rectangles. Circles represent the common theme. (A) Student responses to which aspects of the session were most helpful (question D in Table 1). (B) Student responses to which topics were high priority (question F in Table 1).

Results showed that 100% of students felt teaching was appropriate for their level and felt comfortable asking questions. Moreover, 96% of students felt comfortable answering questions. Overall, 100% of students answered that they would attend a further VTM, and 96% of students would recommend this programme to a friend. Most students found x-ray interpretation most useful, followed by defining specialist terms (Figure 1). Students felt the “breaking down [of] orthopaedic terms into simple terms” increased access to learning. Several students highlighted the benefit of observing “3 doctors talking between themselves and asking and answering questions at a manageable pace and level” (Figure 1). Importantly, one student appraised panel discussion as an “open environment where all feedback was equally entertained”. However, students suggested we use “more menti polls” or “OSCE-focused questions”. Recommendations for future topics converged into two themes: fracture diagnosis/management and interpretation of X-rays (Figure 1). Over 50% of the responses requested fracture content and interpretation of x-rays, and 30% requested “ACL ruptures”, “polytrauma” and “spinal Injuries (Cauda Equina Syndrome)”. A minority requested “paediatric patients” “more anatomy”, “bone healing” physiology, and “musculoskeletal tumours”. The edited VTM was made available on YouTube and the Bristol medical school website (see Table 2 in the appendix). The YouTube account obtained 20 subscribers, with a median of 23 views (±42) per video. The number of views was greater than the number of live attendees per session; this is in keeping with student suggestions to shorten the duration of our live meetings.

## Discussion

COVID-19 has altered the landscape of future medical education, and despite the return to clinical internships, medical schools continue to incorporate virtual strategies into their curricula. The GMC demand rigorous standards from junior doctors and this relies on adequate clinical exposure in medical school [8]. MSK conditions account for >30% of cases in general practice and students require comprehensive coverage of T&O regardless of future specialty choice [9]. Prior to the pandemic, 81% of UK final-year medical students were exposed to orthopaedics and 59% attended trauma meetings [10]. This figure inevitably decreased during the pandemic restrictions, and although virtual solutions have been proposed for many surgical specialties none such as ours have been described for undergraduate T&O education [11-14].

We suggest a long-term, accessible strategy to enhance T&O education in the form of VTM. Students reported a high level of satisfaction and a comfortable learning environment with this method. All the survey responders felt comfortable asking questions and considered the peer interaction between specialists particularly beneficial and inclusive. This supports the well-documented understanding that interactive T&O sessions are most useful to medical students [15,16]. Fracture management and x-ray interpretation were the highest priority topics, the same areas medical students have felt least competent in suggesting our method has merit, and that trauma meetings remain relevant to orthopaedic education [10].

Students expressed high satisfaction, with 96% stating they would attend again and 100% stating they would recommend VTM to a friend. Many students also viewed the meetings on YouTube months later and in greater numbers, possibly because improved accessibility allowed review of challenging areas and increased learner efficiency [17]. The opportunity for asynchronous communication in YouTube’s comment section could also help consolidate difficult material. Interestingly, the number of teaching hours and the satisfaction levels among Scottish orthopaedic trainees increased during the national lockdown and the greatest challenge the NHS workforce has seen in decades [18]. It is possible that VTM could be a strategy that is both student-focused and convenient for clinicians moving forward. We acknowledge however that long-term student interaction with supplementary teaching material is subject to individual initiative, interest, and career aspirations. Therefore, an important determinant of the success of VTM is student interest in orthopaedics, which we did not evaluate. We also acknowledge our sample was small, thus our study may not be generalisable to the average medical student population. Our study was intended to be a proof-of-concept design. A national collaboration between medical schools, could enhance the power, determine the effectiveness of our teaching intervention, and accurately reflect the average student experience.

## Conclusions

This study provides preliminary evidence that VTM are an acceptable learning modality for undergraduates. Student satisfaction, general convenience, and flexibility predict the success of VTM in the foreseeable future. COVID-19 has accelerated the healthcare transition to the virtual world, and it is possible that many more adaptations, such as virtual clinics and operating theatres, are to follow.

## Appendices

**Table 2:** Trauma case based discussion recordings available on Youtube

Trauma case	URLS for edited and recorded virtual trauma meetings (VTM)
Ankle fracture (35y)	https://youtu.be/Z7IdwVU_B68
Forearm fracture (7y)	https://youtu.be/y-u0phMUYLs
Acute knee swelling (68y)	https://youtu.be/nBI8mm-JK8g
Femoral fracture (35y)	https://youtu.be/GkHQdV6NtJg
Wrist fracture (76y)	https://youtu.be/jc6Xhz8dnMU
Neck of femur fracture (94y)	https://youtu.be/Lrg2CxRou3A
Hip fracture (89y)	https://youtu.be/zUy5tsFEC2I
Tibial fracture (33y)	https://youtu.be/yrIudvGx_Po
Open tibial fracture (30y)	https://youtu.be/dXVVWmHtnTU
Supracondylar fracture (10y)	https://youtu.be/nhbGiAdSgyo
